# Long-term gamma transcranial alternating current stimulation improves the memory function of mice with Alzheimer’s disease

**DOI:** 10.3389/fnagi.2022.980636

**Published:** 2022-09-15

**Authors:** Linyan Wu, Tiantian Cao, Sinan Li, Ye Yuan, Wenlong Zhang, Liang Huang, Chujie Cai, Liming Fan, Long Li, Jingyun Wang, Tian Liu, Jue Wang

**Affiliations:** ^1^The Key Laboratory of Biomedical Information Engineering of Ministry of Education, Institute of Health and Rehabilitation Science, School of Life Sciences and Technology, Xi’an Jiaotong University, Xi’an, China; ^2^National Engineering Research Center of Health Care and Medical Devices, Guangzhou, China; ^3^The Key Laboratory of Neuro-informatics & Rehabilitation Engineering of Ministry of Civil Affairs, Xi’an, China

**Keywords:** APP/PS1, hippocampus, transcranial alternating current stimulation, microglia, beta-amyloid

## Abstract

**Background:**

The main manifestation of Alzheimer’s disease (AD) in patients and animal models is impaired memory function, characterized by amyloid-beta (Aβ) deposition and impairment of gamma oscillations that play an important role in perception and cognitive function. The therapeutic effect of gamma band stimulation in AD mouse models has been reported recently. Transcranial alternating current stimulation (tACS) is an emerging non-invasive intervention method, but at present, researchers have not completely understood the intervention effect of tACS. Thus, the intervention mechanism of tACS has not been fully elucidated, and the course of treatment in clinical selection also lacks theoretical support. Based on this issue, we investigated the effect of gamma frequency (40 Hz) tACS at different durations in a mouse model of AD.

**Materials and methods:**

We placed stimulating electrodes on the skull surface of APP/PS1 and wild-type control mice (*n* = 30 and *n* = 5, respectively). Among them, 20 APP/PS1 mice were divided into 4 groups to receive 20 min 40 Hz tACS every day for 1–4 weeks. The other 10 APP/PS1 mice were equally divided into two groups to receive sham treatment and no treatment. No intervention was performed in the wild-type control mice. The short-term memory function of the mice was examined by the Y maze. Aβ levels and microglia in the hippocampus were measured by immunofluorescence. Spontaneous electroencephalogram gamma power was calculated by the average period method, and brain connectivity was examined by cross-frequency coupling.

**Results:**

We found that the long-term treatment groups (21 and 28 days) had decreased hippocampal Aβ levels, increased electroencephalogram spontaneous gamma power, and ultimately improved short-term memory function. The treatment effect of the short-term treatment group (7 days) was not significant. Moreover, the treatment effect of the 14-day treatment group was weaker than that of the 21-day treatment group.

**Conclusion:**

These results suggest that long-term gamma-frequency tACS is more effective in treating AD by reducing Aβ load and improving gamma oscillation than short-term gamma-frequency tACS.

## Background

Alzheimer’s disease (AD) is a neurodegenerative encephalopathy that leads to cognitive deterioration (Scheltens et al., 2021). AD is characterized by cognitive deficits represented by memory dysfunction, and its pathological features are mainly the presence of extracellular senile plaques caused by the accumulation of Aβ (Moreno-Jimenez et al., 2019; Tabassum et al., 2020; Mjwa et al., 2021). Aβ plaques are normally degraded by microglia and astrocytes, but this process is affected as AD progresses. Although medications are currently unable to prevent the accumulation of amyloid plaques in AD patients (Mehta et al., 2017; Cummings et al., 2020), pharmacological and non-pharmacological treatments that induce microglia and astrocytes to degrade Aβ plaques are still the main goal of current research on AD treatments (Abbott and Dolgin, 2016).

Numerous studies have found that neuronal activity at gamma oscillation frequency occurs in multiple brain regions and is speculated to be closely related to cognitive function (Iaccarino et al., 2016). Previous studies have shown that gamma oscillations are generated by synaptic activity between GABAergic inhibitory interneurons and excitatory pyramidal cells (Sohal et al., 2009; Carlén et al., 2012). Significant reductions in spontaneous gamma power and degeneration of gamma synchronization have been observed in both AD patients and AD mouse models (Gillespie et al., 2016; Mably and Colgin, 2018).

Recently, technology based on gamma band entrainment has gradually attracted attention and has become a new method for AD treatment. Studies have shown that gamma stimulation (sound and light) reduces Aβ deposition and increases the number of microglia (Martorell et al., 2019). In the hippocampus and SVZ region of mice with AD, Liu et al. (2020) found that gamma intracranial alternating current stimulation (IACS) can increase Ki67-, Nestin-, and DCX-labeled neurogenesis. However, there is a lack of research on the therapeutic effect of gamma-entrained tACS on AD. Moreover, the stimulatory effects of different durations of tACS in clinical practice are contradictory. In clinical trials, gamma oscillations (30–90 Hz) associated with several higher-order cognitive tasks have been observed to be disrupted in the EEG of AD patients. Gamma stimulation can improve cognitive performance in neurological diseases such as AD by controlling abnormal brain activity (Cirrito et al., 2005; Bero et al., 2011). In a recent study, Hoy found that gamma-tACS improved working memory more than tDCS or sham stimulation (Hoy et al., 2015). This result suggests that gamma-band tACS has the potential to improve AD. Moreover, other studies have shown that short-term tACS did not achieve the desired effect. Clayton used a 30-min alpha-tACS to intervene but did not elicit the desired alpha power (Clayton et al., 2018). Saito found that 3 days of 70 Hz tACS did not improve subjects’ tactile orientation discrimination (Saito et al., 2021). Therefore, the duration of stimulation should be an important factor affecting the effect of gamma-entrained tACS intervention. In this study, we aimed to investigate the tACS intervention technique of the gamma band as a method to reduce brain pathology in a mouse model of AD and determined the effect of different stimulation durations on the effect of tACS intervention.

## Materials and methods

### Experimental animals

**FIGURE 1:**
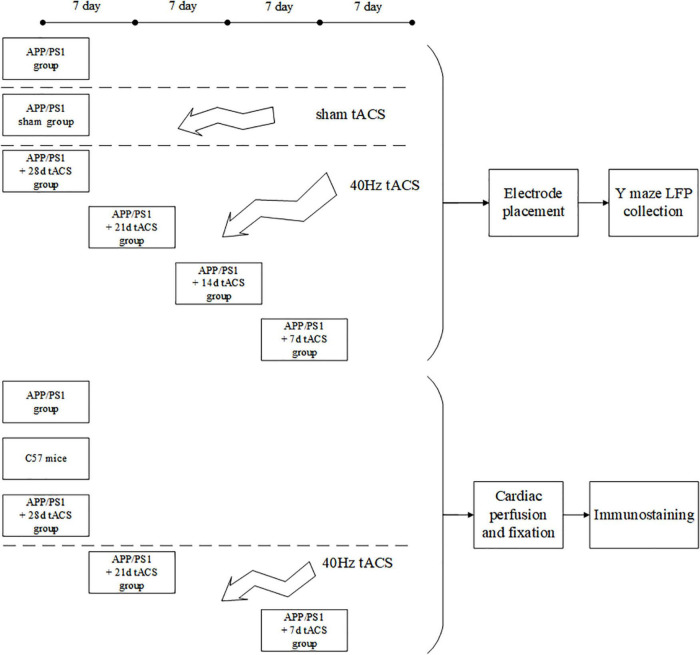
Experimental flow chart.

All the tests in this work were carried out in line with the procedures approved by Xi’an Jiaotong University’s Animal Protection and Use Committee. The animals were kept in a temperature-controlled (22 ± 0.5°C) habitat with a 12-h dark cycle of sunlight and free access to food and drink. Every possible attempt was made to minimize the number of animals utilized and minimize their suffering. APP/PS1 transgenic C57/BL6-Tg mice (Hu APP695swe, PSEN1-d E9) and normal C57/BL6-Tg mice were purchased from the Institute of Medical Experimental Animals, Chinese Academy of Medical Sciences. All mice were 4 months old after the experiment. Because there are studies showing that 4-month-old Alzheimer’s mice have been in Aβ Accumulation stage (Zhou et al., 2017; Dong et al., 2018). The mice in the behavioral and electrophysiological experiments were divided into seven groups: (1) APP/PS1 control, (2) APP/PS1 sham operation, (3) tACS treatment APP/PS1 group (7 days), (4) tACS treatment APP/PS1 group (14 days), (5) tACS treatment APP/PS1 group (21 days), (6) tACS treatment APP/PS1 group (28 days), and (7) C57 mice of the same age group. The immunohistochemical study had three groups: (1) APP/PS1 control group, (2) tACS treatment APP/PS1 group (7 days), and (3) tACS treatment APP/PS1 group (21 days). Five animals were employed in each group. Since electrophysiological experiments can damage the brain, immunohistochemical study requires a complete brain to be sectioned and stained. Therefore, the mice in seven groups for the electrophysiological experiments and three groups for the immunohistochemical experiments were not repeated. In total, there were 50 mice in 10 groups. Figure 1 depicts the experimental procedure.

### Transcranial alternating current stimulation stimulation in APP/PS1 mice

**FIGURE 2:**
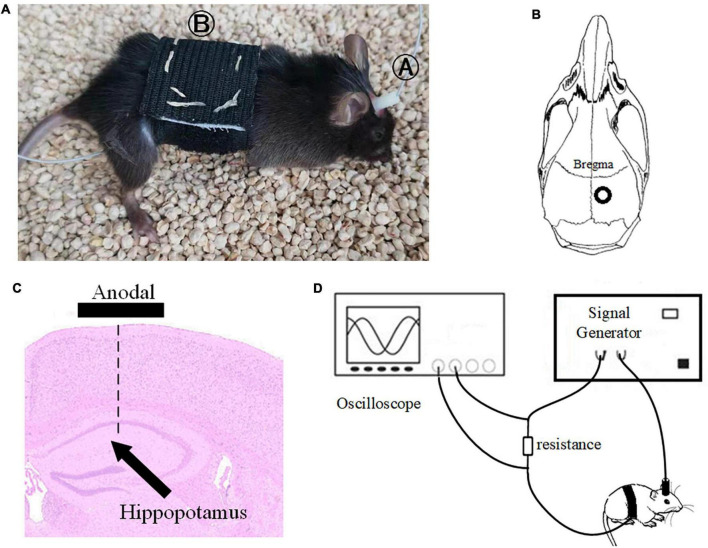
Stimulation diagram. **(A)** Schematic diagram of mouse stimulation. A and B are two electrodes. **(B,C)** Stimulation target. **(D)** We used an oscilloscope to monitor the stimulation current in real time to ensure the efficiency of the stimulation electrode.

Before tACS, the mice were treated with 2% (v/v) isoflurane oxygen anesthesia, the hippocampus position (AP = –2 mm, ML = 1.35 mm) was calculated using a stereotactic apparatus, and the mice were then fixed with 0.8% (v/v) isoflurane oxygen anesthesia. The hair on the head was shaved and sanitized. In the center of the skull, directly above the hippocampus (AP = –2 mm, ML = 1.35 mm), a hollow 3D printing mold was placed and covered with dental base acrylic resin (Figures 2B,C). The printing mold filled with a hydrogel was one stimulation electrode, and another stimulation electrode was placed on the mouse’s chest and abdomen when in use (Figure 2A). The following were the experimental parameters: frequency 40 Hz, amplitude 130 μA (signal created and monitored by a RIGOL DG4202 waveform generator), and stimulation for 20 min every day for 7, 14, 21, and 28 days. The time of tACS performed between groups was the same, and the stimulation current was monitored in real time by an oscilloscope during stimulation to guarantee that the stimulation is stable (Figure 2D). The anesthesia and procedure were the same for the APP/PS1 sham group and the APP/PS1 control group mice. To replicate the switch of the stimulus, we administered a 2 s stimulus with identical parameters solely at the beginning and conclusion of the sham operation group stimulation. The safety of tACS in each group was ensured by monitoring body weight once a day during the scheduled tACS treatment.

### Electrode placement

The APP/PS1 mouse collecting electrodes were implanted shortly after the tACS stimulation period. Mice were anesthetized with isoflurane oxygen at a concentration of 2% (v/v). The mice were then placed on a heating pad that was controlled by a thermostat. The depth of anesthesia in mice was measured in real time, and the amount of anesthetic was raised as needed. The hair on the head was shaved and sanitized. The acquisition electrode was implanted after a craniotomy was conducted directly above the hippocampus (AP = –2 mm, ML = 1.35 mm, DV = 1.4 mm). Screws were inserted above the cerebellum as a reference point for the acquisition electrode. To reduce external electromagnetic interference, we secured the electrode using dental base acrylic resin and encircled it with copper mesh after implantation. For the next 2 days, the mice were assessed twice a day and given caroprofen (2 mg/kg) if they showed signs of pain or stress. The animals were subjected to behavioral tests 2 days after electrode implantation.

### Behavioral analysis

#### Experimental design and procedure

We set up our experimental scheme with reference to the experimental settings of Komorowski et al. (2009) and Tort et al. (2009). We familiarized the mice with the Y-maze setting on the first day. The next day, the experiment formally began to assess the ability of the mice to learn in the Y maze. The mice were placed in any arm of the Y maze during the experiment. Mice were required to enter different arms throughout two consecutive tests. If the mouse starts at arm A and enters arm B for the first time, the second time it must enter arm C instead of arm B. They will be penalized with a 15 V pulse electrical stimulation if they make the wrong choice. The procedure described above is an example of an experiment. The entire experiment was carried out in 1 day to verify the consistency and validity of the gathered EEG data. A total of 90 experiments were carried out for each mouse.

#### Learning curves

The learning curve is a broken line diagram drawn by calculating the average accuracy of the mouse tests every 10 times. The learning curve was generated after 90 experimental records by computing the accuracy in each window through a sliding window with a total length of 10 and step of 5.

### Electrophysiological analysis

All of the analyses were carried out in MATLAB using built-in and custom-written functions. LFP was recorded on the same day after the end of stimulation in all groups, and the sampling rate was 500 Hz. The data were detrended fluctuations before analysis.

#### Spectral analyses, filter settings, and amplitude and phase time series extraction

The power spectrum of the electrophysiological data of mice in the Y-maze selection area was estimated using the Cerebus NeuroExplorer program with a time window of 0.1 s steps. The electrophysiological data of this experiment were then chosen from a time period free of power frequency noise and low frequency interference. The gamma band was filtered by 30–45 Hz filters, while the theta band was filtered by 8–12 Hz filters. Averaging all power spectrum data across the whole time period yielded the average power spectrum estimation result.

#### Modulation index and the phase-amplitude comodulogram

**FIGURE 3:**
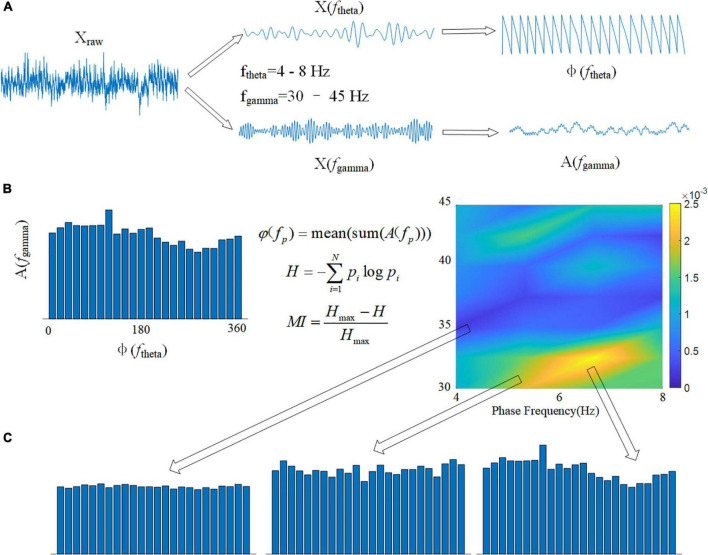
Calculation flowchart for MI. **(A)** The original signal X_raw_ is filtered in two frequency bands, and the phase of the theta band φ(*f*_theta_) and the amplitude of the gamma band *A*(*f*_gamma_) are calculated using the Hilbert transform. **(B)** Using the modulation index formula φ(*f*_p_), calculate the average value of all amplitudes *A*(*f*_*gamma*_) corresponding to each 15° phase in boxes. The spectrum of MI can be drawn using 1 Hz as the phase frequency step and 2 Hz as the amplitude frequency step. **(C)** The variance of MI is represented by the depth of color in the spectrum.

Cross frequency coupling (CFC) is a measurement of the interaction between LFPs in multiple frequency bands and is used to evaluate memory function (Tort et al., 2013). The intensity of theta-gamma phase-amplitude coupling was measured using the modulation index (MI). To obtain a slow (theta, 8–13 Hz) and a fast (gamma, 30–42 Hz) frequency range of interest, we independently filtered the LFP twice. Theta- and gamma-filtered signals were then used to obtain phase (theta) and amplitude (gamma) time series, respectively (Figure 3A). The mean amplitude of each phase bin was computed using a combined time series made up of instantaneous phase vs. amplitude values. The intensity of phase-amplitude coupling between the theta phase and gamma amplitude was then measured using the MI (Figure 3B). Finally, by applying this framework to several frequency pairs and presenting the related MI values as a heatmap in a bidimensional plot (Figure 3C; Tort et al., 2008).

### Pathology and immunofluorescence analysis

#### Brain tissue fixation and sectioning

Euthanized mice (five mice in each group) were perfused and preserved with frozen 0.1 MB solution and 4% PFA. The brain was then dissected and incubated for 3 days in a 4% PFA solution at 4°C before being transferred to a 30% (v/v) sucrose solution at 4°C for an additional 3 days of incubation. The brain was frozen with dry ice after cryopreservation, and coronal sections were made every 40 μm with a frozen slicer. Coronal brain sections were collected between AP + 1 and – 0.5 mm (thickness 1.5 mm) from bregma (including the SVZ) and between AP – 1.2 and – 2.7 mm (thickness 1.5 mm) from the bregma (including the hippocampus).

#### Immunofluorescence

The brain slices were fixed for 30 min in 4% PFA and then infiltrated for 30 min with 0.1% Triton X-100 (Sigma-Aldrich). The non-specific protein was sealed for 1 h at room temperature with 3% bovine serum albumin (BSA) in 0.1 M phosphate buffered saline (PBS) solution and then incubated with antibodies: anti-Iba1 (1:400, ab178846, Abcam) and anti-amyloid (1:200, 13075SS, Novus) at 4°C overnight. After the slices were rinsed with PBS, they were transferred to a 3% BSA-PBS solution containing goat anti-mouse/rabbit (Alexa Fluor 594/488, 1:1000, A11005, A-11034, Invitrogen) secondary antibodies. We used 4’,6-diamidino-2-phenylindole (DAPI) to indicate the nuclei. A high-resolution digital pathological scanning system (3D HISTECH Pannoramic MID II with ×20, ×40, and ×63 objectives) was used to detect fluorescence. AD has been proven to increase Aβ deposition and neuroinflammation (Um et al., 2012; Chakrabarty et al., 2015). Inflammation and Aβ deposition have been proven to be eliminated by microglia (Pascoal et al., 2021). Therefore, these markers were employed to study AD mouse neurons and Aβ deposition.

### Statistics

#### Local field potential analysis

Univariate ANOVA was used to examine the MI and gamma band power spectrum between the two groups. The power spectrum data were examined with the LSD hypothesis equivariance test after the homogeneity test, and the MI data were tested with the Tamheni T2 non-hypothesis equivariance post-test. In these studies, a confidence level of *p* < 0.05 was employed.

#### Quantification of immunopositive cells

CaseViewer software, ImageProPlus software and ImageJ software were used to count positive cells of the hippocampus. With a × 40 objective lens, Iba1 + cells and Aβ were quantified in each region. We chose three perspectives with a total size of 800 × 300 μm^2^ for each section’s cell count. Iba1 immunohistochemistry were converted to binary and skeletonized images using ImageJ. The Analyze Skeleton Plugin was used to measure microglial ramification. The number, area and body diameter of microglia were obtained by counting and calculating the branches, junctions and endpoints of microglia after skeletonization. The average value and standard deviation of the number of cells in each group’s hippocampal area were calculated.

SPSS software was used to analyze the data, which followed the general linear model. The alpha level of type I error was set to 0.05 to reject the erroneous hypothesis. The information is presented as the mean standard deviation (SEM). One-way ANOVA was used to compare the number of neurogenic cells stained by Iba1 in each group, the sedimentary area of Aβ in each group and the diameter of Iba1-positive microglial cell bodies.

## Results

### Mouse behavior improved after gamma transcranial alternating current stimulation

**FIGURE 4:**
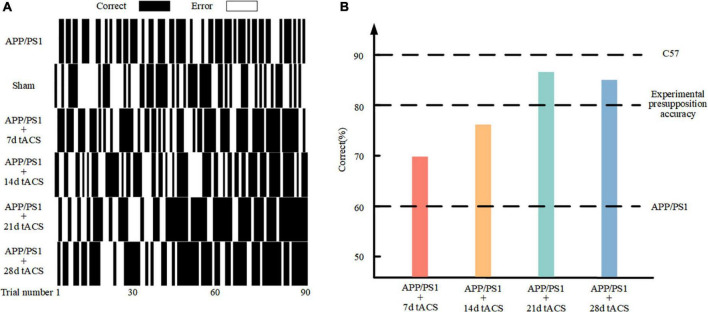
**(A)** Behavioral results in the Y maze. **(B)** Comparison of 90 behavioral results in the different stimulation time groups.

Figure 4A depicts the behavior of various groups of mice as it improved over time during the learning process. The correct rate of mice with senile dementia with electrical stimulation increased throughout the trial (black is correct and white is incorrect). The behavioral comparison of the 7-day stimulation, 14-day stimulation, 21-day stimulation, 28-day stimulation, and control mice is depicted in Figure 4B. The mice with AD could only achieve a 60% correct rate in 90 rounds of training, while the normal mice could achieve a 90% correct rate. We used 80% accuracy as the threshold to judge the short-term memory function of the mice. The mice with AD with 21 and 28 days of gamma tACS could complete the training of memory tasks within 80 days, which was better than that of the short-term stimulation group (7 and 14 days).

### Spontaneous gamma power and cross frequency coupling value in local field potential changed after gamma transcranial alternating current stimulation

**FIGURE 5:**
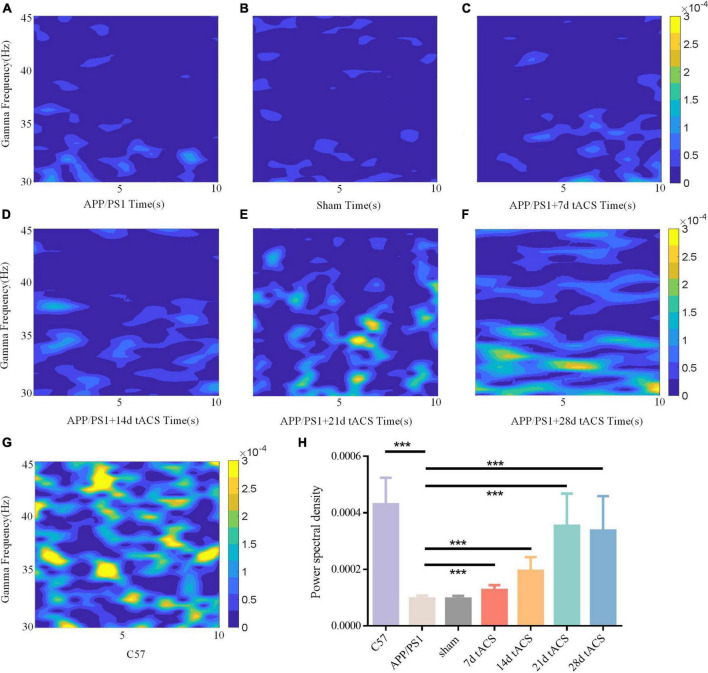
**(A)** APP/PS1 group power spectrum, **(B)** sham group power spectrum, **(C)** 7-day APP/PS1 group power spectrum, **(D)** 14-day APP/PS1 group power spectrum, **(E)** 21-day APP/PS1 group power spectrum, **(F)** 28-day APP/PS1 group power spectrum, **(G)** C57 group power spectrum. **(H)** Spontaneous gamma power between different groups, * indicates *P* < 0.05, ** indicates *P* < 0.01. *n* = 5 mice for each group. The power spectral density of C57 group was 0.000433 ± 0.000127, APP/PS1 group was 0.000092 ± 0.000035, sham group was 0.00009 ± 0.000015, 7 days tACS group was 0.000138 ± 0.000055, 14 days tACS group was 0.000204 ± 0.000072, 21 days tACS group was 0.000377 ± 0.000141, and 28 days tACS group was 0.000364 ± 0.000153.

In the Y maze, the LFP of all groups in the hippocampus region was measured, and the changes in gamma frequency following gamma stimulation were examined. The gamma band LFP power spectral estimation of seven groups of mice with AD is shown in Figures 5A–G. We confirmed the effectiveness of the gamma band stimulation effect by ANOVA analysis. The LFP of the gamma band in the hippocampus region of mice with AD has been found to be lower than that of normal mice, and our results also verify this conclusion (Li et al., 2012; Mondragón-Rodríguez et al., 2018). There was a significant difference in power spectrum between C57 group and APP/PS1 group (*p* = 0.002). We found that with increasing stimulation time, gamma tACS improved the spontaneous gamma power spectrum (Figure 5H). The p value of 7-day tACS group was 0.041, decreased to 0.022 in 14-day tACS group, and reached 0.004 (21-day tACS group) and 0.005 (28-day tACS group).

**FIGURE 6:**
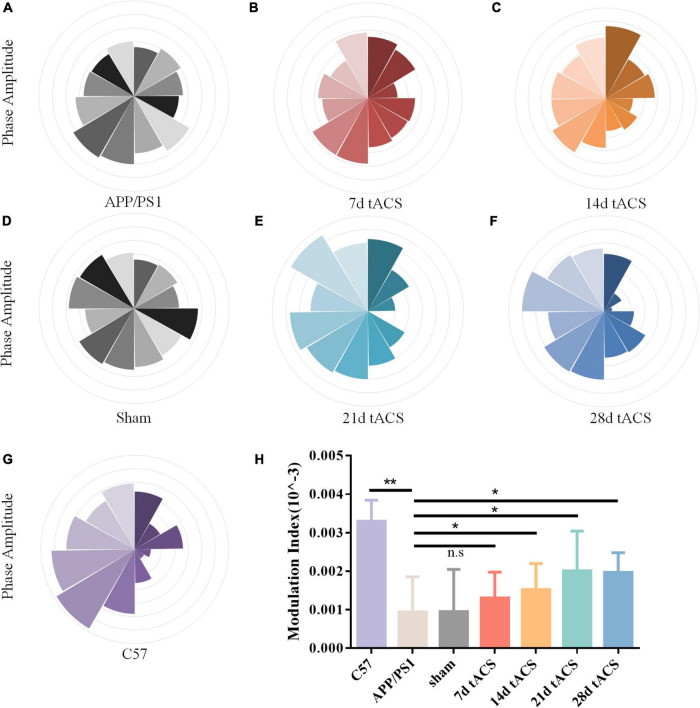
**(A)** APP/PS1 group MI spectrum, **(B)** 21 days APP/PS1 group MI spectrum, **(C)** 28 days APP/PS1 group MI spectrum, **(D)** sham group MI spectrum, **(E)** 21 days APP/PS1 group MI spectrum, **(F)** 28 days APP/PS1 group MI spectrum, **(G)** C57 group MI spectrum, **(H)** MI of CFC between different groups, * indicates *P* < 0.05, ** indicates *P* < 0.01. *n* = 5 mice for each group. The MI index of mice in C57 group was 0.0033 ± 0.0006, the MI index of mice in APP/PS1 group was 0.0009 ± 0.0009, the MI index of mice in sham group was 0.001 ± 0.0011, the MI index of mice in 7 days tACS group was 0.0013 ± 0.0025, the MI index of mice in 14 days tACS group was 0.0016 ± 0.0006, the MI index of mice in 21 days tACS group was 0.0021 ± 0.0011, and the MI index of mice in 28 days tACS group was 0.0020 ± 0.0005.

We computed the MI of these groups. Similar to the power spectrum results, gamma tACS improved the CFC in the mice with AD. The difference between the WT group and the APP/PS1 group was *p* = 0.008, and there was no difference between the 7-day group. The *p*-value of the 14-day tACS group and the APP/PS1 group was 0.037, the *p*-value of the 21-day tACS group and the APP/PS1 group was 0.014, and the *p*-value of the 28-day tACS group and the APP/PS1 group was 0.019. With increasing stimulation time, the recovery effect of tACS on cross-frequency coupling was enhanced (Figure 6). Moreover, the effect of 21 days of stimulation and 28 days of stimulation was at the same level.

### Aβ loads in the hippocampus changed after long-term gamma transcranial alternating current stimulation

**FIGURE 7:**
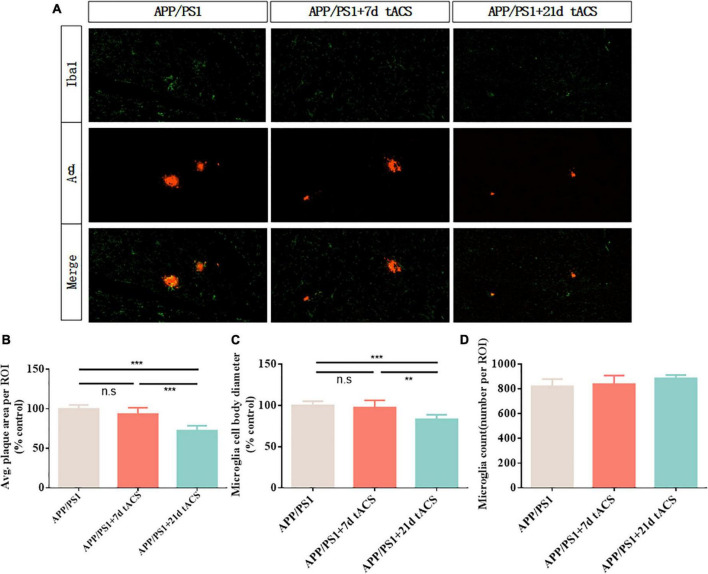
Transcranial alternating current stimulation induces a microglial response in the hippocampus of APP/PS1 mice. **(A)** Immunohistochemistry with anti-Iba1 and anti-Aβ antibodies in the hippocampus of APP/PS1 mice. **(B)** Average area of Aβ-positive plaques in the hippocampus. **(C)** Diameter of Iba1-positive microglial cell bodies in the hippocampus. **(D)** Number of Iba1-positive microglia in the hippocampus. ** Indicates *P* < 0.01,*** indicates *P* < 0.001. *n* = 5 mice for each group. The marks in the diagram represent data points. The Aβ area of mice in 7 days tACS group was 96% ± 2.1% of that of APP/PS1 group. The Aβ area of mice in 21 days tACS group was 77% ± 6.9% of that of APP/PS1 group. The microglia diameter of mice in 7 days tACS group was 98% ± 1.6% of that of APP/PS1 group. The microglia diameter of mice in 21 days tACS group was 81% ± 5.3% of that of APP/PS1 group.

We discovered that long-term stimulation for more than 21 days had a better effect than short-term stimulation for 7 and 14 days, based on earlier findings (Figure 7A). We chose 7-day stimulation as the representative time for short-term stimulation since the results of the 7-day stimulation were similar to those of the 14-day stimulation in earlier investigations. Based on the same principle, we chose 21 days of stimulation as the representative of long-term stimulation. In the hippocampus of the 21-day tACS groups, deposition was considerably reduced compared to that in the APP/PS1 group (*p* = 0.003). Moreover, a decrease in deposition was not visible in the hippocampus of the 7-day stimulation group (Figure 7B). In the 21-day tACS groups, although there was no significant difference in the number of microglia, the cell body diameter of microglia decreased (*p* = 0.004). The morphology of activated microglia was higher in the 7-day stimulation group than in the 21 stimulation groups (*p* = 0.008) (Figures 7C,D). There was no statistically significant difference in Aβ deposition or microglia between the 7-day stimulation group and the APP/PS1 stimulation group.

## Discussion

Abnormal neural signal oscillations and increased Aβ deposition have been documented in studies of human AD brains and animal models (Griciuc et al., 2013). We tested gamma tACS for different durations in AD mouse models and observed the relationship between stimulation time and stimulation effect. Our recordings showed that no weight loss or neurological impairment occurred during the experiment, suggesting that tACS is a safe treatment. We present new evidence that, compared with short-term gamma-band tACS, long-term (over 21 days) gamma-band tACS can better reduce the development of AD-associated pathology in mice and restore abnormal gamma oscillations, thereby improving cognitive function in mice with AD. The novelty of our method is the application of tACS at 40 Hz and the identification of the optimal course of treatment to achieve efficacy and safety.

### Long-term gamma transcranial alternating current stimulation improves behavior of mice with Alzheimer’s disease in the Y maze

Studies have shown that the memory function of 4-month-old APP/PS1 mice is attenuated and can be detected behaviorally in the Y maze or water maze (Liu et al., 2007). To investigate the effect of tACS on memory function in the mice with AD, we selected 3-month-old APP/PS1 mice to analyze their behavioral improvement after one month of tACS stimulation. Tot studied the short-term memory ability of normal rats in the Y-maze and found that normal rats could achieve 90% selection accuracy in 80 trials (Tort et al., 2009). We performed Y-maze experiments on normal mice and found that normal mice were also 90% correct in our experimental setup. Considering that the memory function of AD patients usually does not recover to the same degree as that of healthy people after electrical stimulation intervention (Lane et al., 2018), we reduced the selection accuracy to 80% to judge whether the memory function of the mice with AD recovered after intervention. In our study, we observed that although the memory function of the mice with AD was improved after gamma tACS stimulation, only long-term gamma tACS stimulation could restore the short-term memory function of the mice with AD to a better level. We speculated that the behavioral improvements were associated with improvements in amyloid-β (Aβ) and gamma oscillations in the mouse brains and conducted subsequent studies.

### Transcranial alternating current stimulation stimulation can entrain gamma band oscillations

**FIGURE 8:**
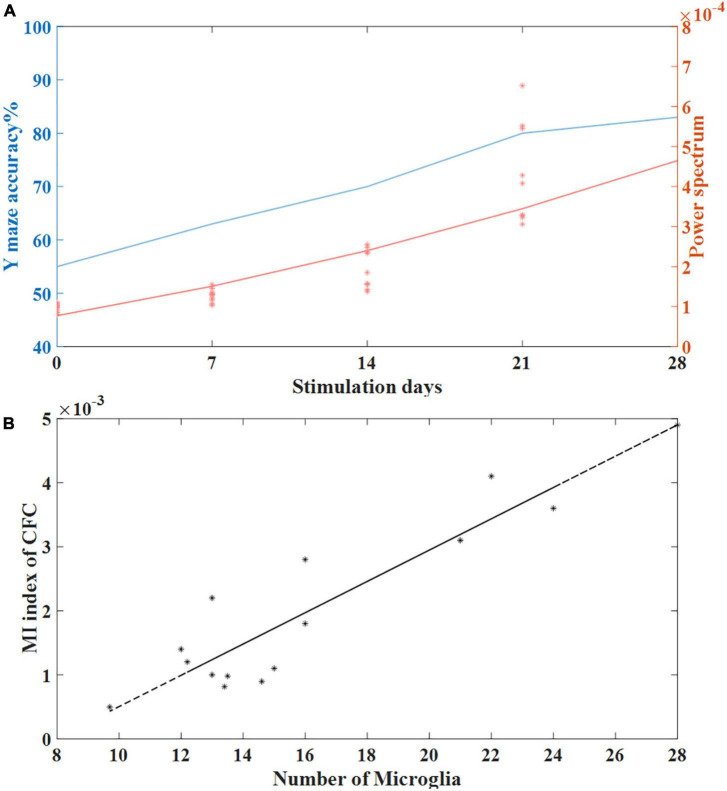
**(A)** The relationship between stimulation time and behavioral accuracy and the relationship between stimulation time and spontaneous gamma power spectrum. **(B)** Correlation between the number of microglia and the MI index of CFC in the tACS treatment groups (*n* = 20). The line is the best-fit linear regression (stars), with correlation *r* = 0.92 (Pearson, *P* < 0.01).

Fast-spiking parvalbumin (PV) cells are GABAergic interneurons that receive NMDA excitatory input from pyramidal cells (Jones and Bühl, 1993). Studies have shown that modulation of fast-spiking PV interneurons through GABAergic inhibitory synaptic activity onto excitatory pyramid cells can generate and fine-tune gamma oscillations in the brain (Sohal et al., 2009; Carlén et al., 2012). Gamma oscillations in the brain play an important role in perception and cognition, and previous studies have shown that cognitive impairment in AD patients is closely related to the reduction of gamma oscillations in the brain (Koenig et al., 2005; Verret et al., 2012). Verret et al. (2012) reported that restoring gamma activity by improving PV cells can reduce cognitive loss in AD animal models (Tort et al., 2013). In our study, we observed that tACS at 40 Hz can entrain gamma-band oscillations. We found that the stimulation time of gamma tACS was positively correlated with the induced power spectrum. The longer the stimulation time, the higher the gamma power spectrum of the mice with AD was and the smaller the gap between the mice with AD and normal mice was. Correspondingly, the longer the stimulation time was, the better the recovery of short-term memory function in the mice with AD was. We calculated coherence plots of stimulation time with correctness and gamma power spectrum (Figure 8A) and found that stimulation time was positively correlated with both gamma power spectrum and behavioral correctness with similar trends.

### Functional improvement can be achieved by long-term transcranial alternating current stimulation

Recent studies have reported that cognitive impairment in AD patients is also manifested in the loss of EEG synchronization (Koenig et al., 2005). Because our studies have shown that tACS at 40 Hz can increase spontaneous gamma power, we further investigated whether tACS could induce functional improvements in the APP/PS1 mouse model. Cross-frequency coupling is considered a useful tool for assessing cognitive function (Belluscio et al., 2012). Analyzing the coupling across frequencies can determine how the phase of low-frequency oscillations modulates high-frequency power, especially in the gamma band. Recent studies have shown that theta-phase gamma coupling is related to working memory by manipulating the order of information during working memory (Jo et al., 2014). Bamberger et al. (2003) reported a link between theta-gamma coupling and memory deficits. Theta-gamma coupling has also been considered a biomarker for assessing memory-related network activity (Heneka et al., 2015). Our results showed that 40 Hz tACS stimulation can increase theta-gamma coupling in the mice with AD compared with the mice in the no-stimulation group, and the effect increased with stimulation time, similar to the improvement in the power spectrum with stimulation time. All these results support that the gamma band produced by tACS stimulation at 40 Hz normalizes cross-frequency coupling, improving brain connectivity and information processing by modulating gamma oscillations in the mouse hippocampus.

### Aβ load decreases with long-term transcranial alternating current stimulation

We speculate that the improvement of spontaneous gamma oscillation in the hippocampus may be related to the reduction of Aβ in the hippocampus of the mice with AD. In fact, Aβ can interfere with neuronal network oscillations by influencing microglia (Ta et al., 2019). Recently, studies have shown that gamma entrainment by optogenetics or visual stimulation influences the phagocytic activity of microglia (Iaccarino et al., 2016). Microglia can be pathogenic or therapeutic (Erkhratsky and Nedergaard, 2018; Park et al., 2021), but unfortunately, our histological marker, Iba1, cannot differentiate between subtypes of microglia. We speculate that the activated microglia in the experiments imply a shift in the microglial phenotype from a proinflammatory M1 subtype to an anti-inflammatory M2 subtype. Namely, tACS may reduce insoluble Aβ in the hippocampus of the mice with AD through the phagocytic activity of M2 microglia and improve gamma oscillations by reducing neuroinflammation.

Compared with those of the no-stimulation group, Aβ levels in the hippocampus decreased after prolonged (21 days, 28 days) tACS, whereas there were no significant changes in Aβ levels in the short-term (7 days) stimulated hippocampus. Therefore, tACS showed a therapeutic effect in terms of Aβ levels. Amyloid plaques formed by the accumulation of Aβ are a pathological hallmark of AD (Brown et al., 2013) and lead to neurite damage, which is causally related to neuroinflammation in APP/PS1 transgenic mice (Meyer-Luehmann et al., 2008; Heneka et al., 2015). In fact, our histological analysis showed that the prolonged tACS treatment was able to better reduce the area of Aβ plaques and decrease cell body diameter of microglia, implying better activation of microglia (Morrison et al., 2017).

Microglia, as important players in synaptogenesis, is closely related to neurotransmission (Verkhratsky and Nedergaard, 2018). Grigorovsky (2018) found that microglia regulate cross-frequency coupling of electrical rhythms in the brain through synaptic effects. We used the cross-frequency coupling coefficient as a marker of gamma electrical rhythm to conduct a correlation analysis with the length of microglial processes and found that the cross-frequency coupling coefficient was negatively correlated with the length of microglial processes. This finding means that compared to short-term tACS stimulation, long-term tACS activates microglia by reducing the number of Aβ plaques and decreasing the length of microglial processes to better restore abnormal gamma oscillations in the hippocampus (Figure 8B).

### Long-term gamma transcranial alternating current stimulation is better than short-term gamma transcranial alternating current stimulation

**FIGURE 9:**
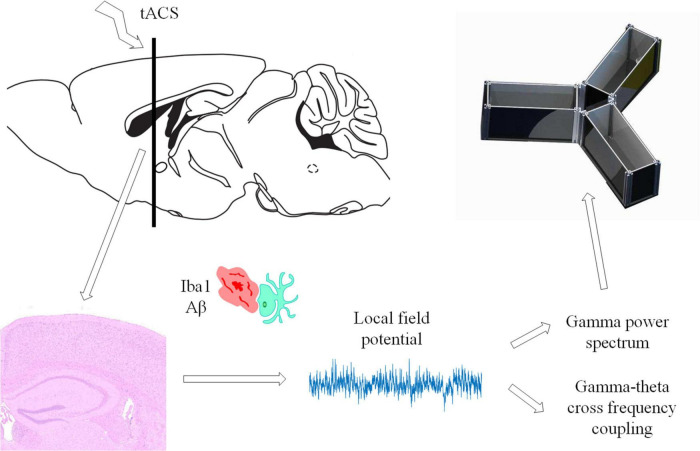
Summary diagram showing that long-term tACS improves the memory function of AD mice.

Khatoun et al. (2017) noted that the results of long-term stimulation and short-term stimulation are different. Mincheol indicated that for 40 Hz band ultrasound stimulation, the 14-day stimulation effect was much better than the 7-day stimulation effect (Park et al., 2021). We found that short-term (7 days) gamma-tACS could not reduce the influence of microglia by Aβ and could not interfere with gamma connections in the brain. The increase in the gamma power spectrum of physiological signals in the brain was more likely to be the result of direct coupling of gamma current to the brain LFP. It is possible that 7 days of tACS cannot fundamentally interfere with AD in mice. Long-term gamma tACS can better reduce Aβ deposition and activate microglia, restore normal gamma oscillations in the brain, and improve short-term memory function in AD mice (Figure 9). On the other hand, tACS is considered to achieve the intervention of AD through modulation of spike timing dependent plasticity and enhancing synaptic plasticity (Di Lorenzo et al., 2018; Vogeti et al., 2022). These biomarkers are considered to predict the clinical progression to dementia in patients with memory impairment of AD (Di Lorenzo et al., 2020). We speculate that long-term continuous tACS may help to enhance the post effect of stimulation, so as to make Alzheimer’s mice recover from cognitive impairment in behavior after stimulation. However, we speculate that the stimulation time is not as long as possible. Although both 21 and 28 days of gamma tACS stimulation showed significant improvements, the effects of 21 and 28 days of stimulation were not significantly different from each other from the perspective of Aβ and intracerebral gamma oscillations. We speculate that prolonged tACS may cause fatigue in mice. Moreover, our experimental paradigm was continuous stimulation for 3–4 weeks rather than stopping stimulation for 1–2 days per week, as in some experimental settings. This method may be the reason why the intervention effect of the 4-week stimulation did not increase significantly compared with that of the 3-week stimulation.

## Conclusion

In the present study, gamma tACS decreased Aβ levels in the hippocampus of APP/PS1 mice and increased spontaneous gamma power and CFC, indicating functional improvement. The stimulation effect of gamma tACS is related to the length of stimulation time, and long-term gamma tACS can obtain a better effect. In conclusion, tACS brain stimulation at 40 Hz could be a potential therapeutic modality by reducing Aβ load and improving brain connectivity. In future studies, the exact neurobiological mechanisms of these effects need to be investigated.

## Code availability

Custom Matlab code that was used to analyze is available from the corresponding author upon request.

## Data availability statement

The original contributions presented in this study are included in this article/Supplementary material, further inquiries can be directed to the corresponding authors.

## Ethics statement

The animal study was reviewed and approved by Xi’an Jiaotong University’s Animal Protection and Use Committee.

## Author contributions

LW, TL, and JW designed the ferret experiments. LW, TC, WZ, and LH programmed the experiment. LW, TC, WZ, CC, JYW, and LH acquired the electrophysiological data for experiment. LW, YY, LF, and LL acquired the cell biology data for experiment. LW and SL analyzed the data. All authors wrote the manuscript.

## Abbreviations

*A*β: beta-amyloid
AD: Alzheimer’s disease
CFC: cross frequency coupling
DCX: doublecortin
IACS: intracranial alternating current stimulation
LFP: local field potential
MI: modulation index
tACS: transcranial alternating current stimulation.
